# A Therapeutic Dilemma: Catastrophic ST‐Elevation Myocardial Infarction Following Antiplatelet Therapy Interruption in a Patient With JAK2‐Positive Polycythemia Vera

**DOI:** 10.1002/ccr3.73264

**Published:** 2026-07-29

**Authors:** Behrouz zarei, Amirhossein Rezaei Latifi, Mohammad Salari

**Affiliations:** ^1^ Department of Cardiology, Faculty of Medicine Hormozgan University of Medical Sciences Bandar Abbas Iran; ^2^ Student Research Committee Hormozgan University of Medical Sciences Bandar Abbas Iran

**Keywords:** bridging therapy, case report, dual antiplatelet therapy, polycythemia vera, recurrent myocardial infarction

## Abstract

Polycythemia vera is associated with a thrombotic–hemorrhagic paradox. We report a 75‐year‐old woman with recurrent ST‐elevation myocardial infarction and stent thrombosis after premature dual antiplatelet therapy interruption. Revascularization was successful, and eptifibatide bridging enabled safe surgery. This case highlights the risks of interruption and the role of bridging therapy.

## Key Clinical Message

This case highlights the thrombotic–hemorrhagic paradox in polycythemia vera and demonstrates that interruption of antiplatelet therapy can trigger catastrophic stent thrombosis, whereas bridging therapy with short‐acting intravenous antiplatelet agents may offer a safer perioperative alternative in carefully selected high‐risk patients.

## Graphical abstract

Premature interruption of dual antiplatelet therapy in a patient with polycythemia vera and clinically suspected acquired von Willebrand syndrome triggered catastrophic recurrent stent thrombosis, whereas perioperative bridging with eptifibatide enabled safe surgery.

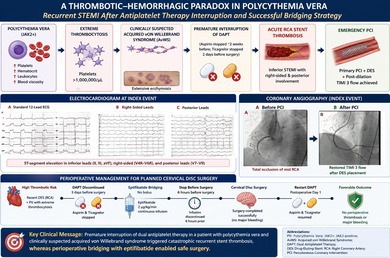

## 1 Introduction

Acute coronary syndrome (ACS) in patients with myeloproliferative neoplasms (MPNs), particularly polycythemia vera (PV), represents a complex and high‐risk clinical entity. This spectrum includes ST‐elevation myocardial infarction (STEMI), non–ST‐elevation myocardial infarction, and unstable angina. The markedly increased cardiovascular risk in these patients is driven by extreme thrombocytosis, leukocytosis, increased blood viscosity, and activating JAK2 mutations. As a result, cardiovascular events account for up to 76% of mortality in this population [1, 2].

Paradoxically, severe thrombocytosis may predispose patients to bleeding rather than thrombosis. When platelet counts exceed 1,000,000/μL, excess circulating platelets promote shear‐induced proteolysis of high–molecular‐weight von Willebrand factor (vWF) multimers, leading to acquired von Willebrand syndrome (AvWS), most commonly type 2A. This results in impaired platelet adhesion, prolonged bleeding time, and mucocutaneous hemorrhages such as spontaneous ecchymosis, despite an underlying prothrombotic milieu [3, 4].

Management becomes particularly challenging in patients with PV who also have advanced coronary artery disease requiring percutaneous coronary intervention (PCI). Dual antiplatelet therapy (DAPT) is essential for the prevention of stent thrombosis, yet bleeding complications may necessitate treatment interruption. Evidence guiding perioperative antiplatelet management in patients with extreme thrombocytosis remains limited, and no randomized trials define optimal strategies in this setting [5, 6, 7, 8, 9, 10, 11].

## 2 Case History/Examination

A 75‐year‐old woman with a history of JAK2‐positive polycythemia vera and advanced coronary artery disease was admitted to our center with acute chest pain. Polycythemia vera had been diagnosed two years earlier, and cytoreductive therapy with hydroxyurea at a dose of 500 mg daily was initiated. However, the patient reported poor adherence to pharmacologic therapy and hematology follow‐up during the year preceding the index event.

Her medical history was notable for multiple prior myocardial infarctions and repeated coronary interventions. In March 2023, she presented with acute ST‐elevation myocardial infarction and underwent successful percutaneous coronary intervention (PCI) of the left anterior descending (LAD) artery with implantation of a 2.5 × 24 mm drug‐eluting stent (DES). Post‐dilation was subsequently performed to optimize stent expansion. In March 2024, due to progression of coronary artery disease, the patient underwent staged PCI of the proximal and distal right coronary artery with implantation of two drug‐eluting stents measuring 2.75 × 18 mm in the proximal RCA and 2.5 × 24 mm in the distal RCA, resulting in complete revascularization with final TIMI 3 flow. Post‐dilation was subsequently performed to optimize stent expansion. On September 10, 2025, she underwent coronary angiography because of recurrent angina. The angiogram demonstrated significant left main coronary artery disease with concomitant two‐vessel disease. Coronary artery bypass grafting (CABG) was strongly recommended. However, the patient declined surgical revascularization. Subsequently, on September 18, 2025, PCI of the left main coronary artery extending into the LAD was successfully performed using a 3.5 × 21 mm drug‐eluting stent, resulting in complete revascularization with TIMI 3 flow. Post‐dilation was subsequently performed to optimize stent expansion. Following the procedure, dual antiplatelet therapy with aspirin and ticagrelor was continued. Approximately one month later, on October 19, 2025, she presented with acute inferior ST‐elevation myocardial infarction. Emergency coronary angiography revealed acute thrombosis of the RCA, and primary PCI was performed with implantation of a 3.0 × 20 mm drug‐eluting stent, achieving complete revascularization without procedural complications. Post‐dilation was subsequently performed to optimize stent expansion.

In January 2026, approximately two weeks before the current myocardial infarction, aspirin (80 mg daily) was discontinued by the treating hematologist after the patient developed extensive, diffuse cutaneous ecchymosis in the setting of extreme thrombocytosis. Laboratory evaluation at that time revealed a platelet count of 1,155,000/μL, a hemoglobin level of 15.2 g/dL, and a white blood cell count of 16,000/μL. Given the presence of JAK2‐positive polycythemia vera, extreme thrombocytosis, and clinically significant bleeding manifestations, clinically suspected acquired von Willebrand syndrome (AvWS) was considered. However, confirmatory laboratory testing was not performed due to clinical, technical, and logistical limitations. The bleeding manifestations did not require hospitalization, blood transfusion, or invasive intervention and were managed conservatively in the outpatient setting.

Two days prior to presentation, ticagrelor (90 mg twice daily) was also withheld in preparation for a planned neurosurgical procedure, despite explicit cardiology recommendations to continue dual antiplatelet therapy. Following interruption of antiplatelet therapy, the patient developed acute chest pain and was diagnosed with an ST‐elevation myocardial infarction on the standard 12‐lead electrocardiogram shown in Figure 1A, with additional right ventricular involvement demonstrated on the right‐sided electrocardiogram in Figure 1B, and posterior involvement on the posterior electrocardiogram in Figure 1C. Urgent coronary angiography demonstrated a culprit lesion in the mid right coronary artery (Figure 2A). Primary PCI was successfully performed with implantation of a 2.5 × 28 mm drug‐eluting stent, resulting in complete revascularization with 0% residual stenosis and restoration of TIMI 3 flow without procedural complications. Post‐dilation was subsequently performed to optimize stent expansion (Figure 2B). The patient's clinical condition improved following revascularization and reinitiation of appropriate medical therapy.

**FIGURE 1:**
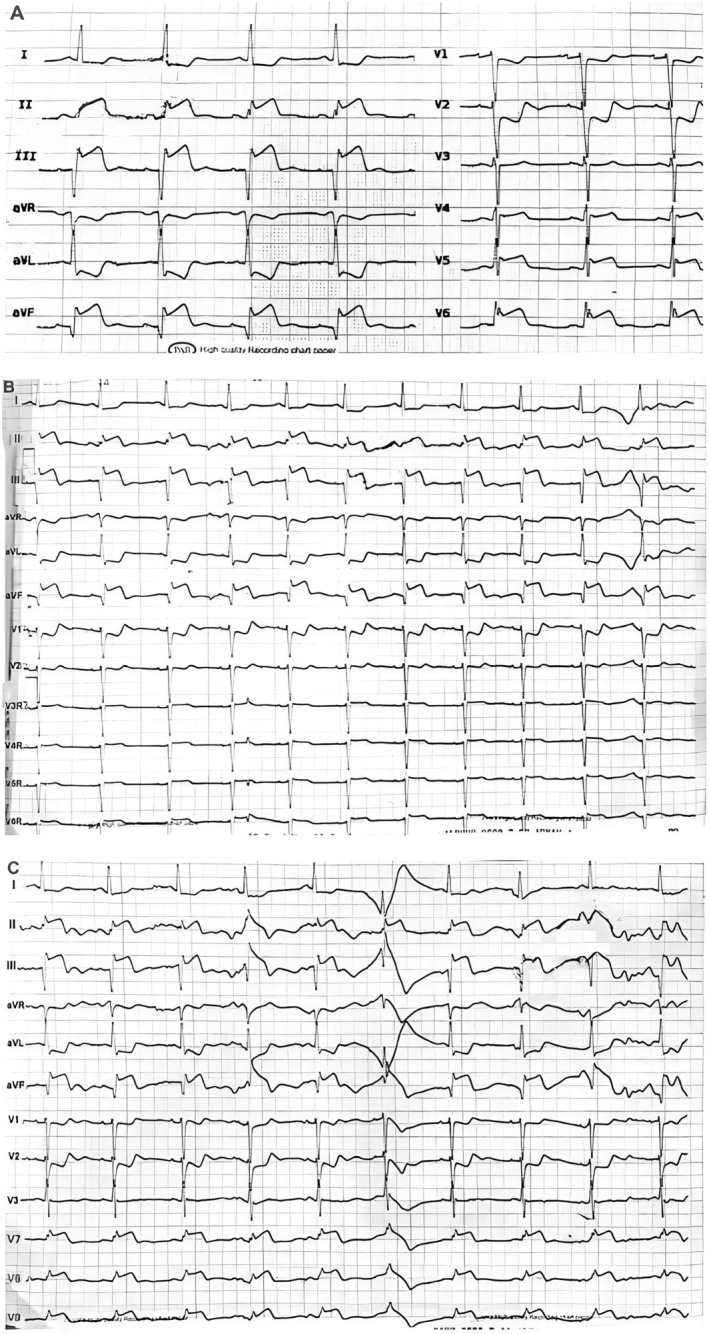
Electrocardiographic findings during the index inferior ST‐elevation myocardial infarction. (A) standard 12‐lead electrocardiogram demonstrating acute inferior STEMI. (B) Right‐sided leads confirming extension of infarction. (C) Posterior leads confirming extension of infarction.

**FIGURE 2:**
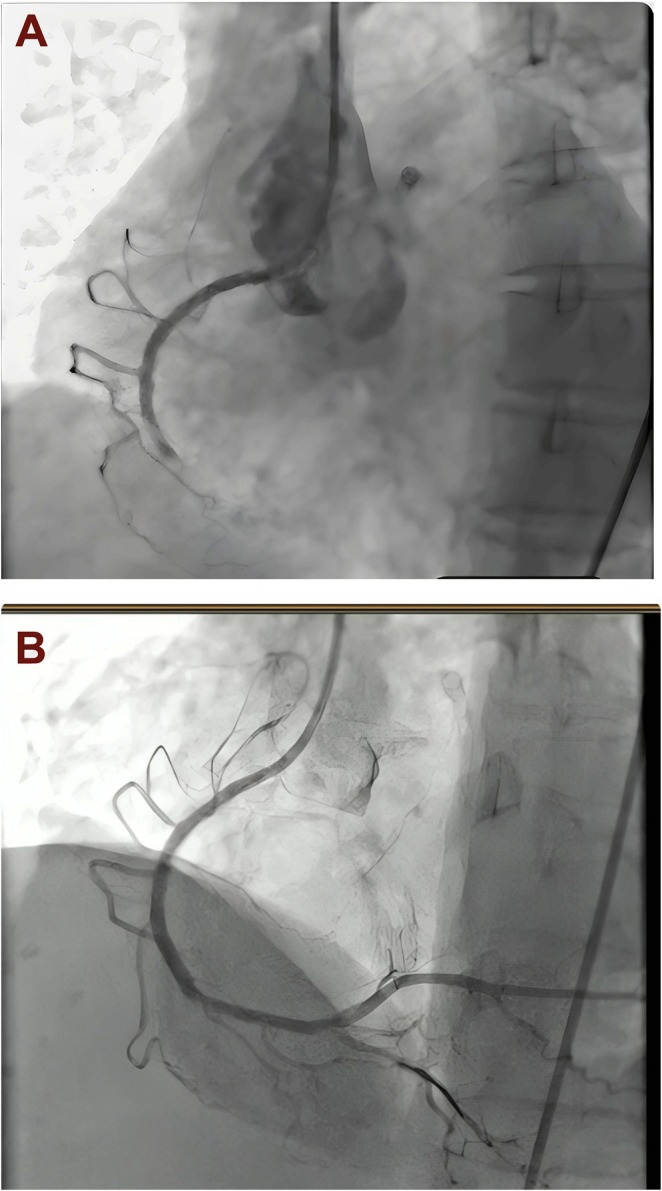
Coronary angiography during index ST‐elevation myocardial infarction. (A) Acute thrombotic culprit lesion involving the mid right coronary artery, with a previously implanted proximal stent visible. (B) Final angiographic result following successful percutaneous coronary intervention with restoration of coronary flow.

In February 2026, due to persistent neurological symptoms, the patient was admitted for the previously planned cervical disc surgery. Given the high thrombotic risk, a bridging strategy was implemented. Aspirin and ticagrelor were discontinued 3 days before surgery, and intravenous eptifibatide, a short‐acting intravenous antiplatelet agent, was initiated without a bolus dose as a continuous infusion at 2 μg/kg/min. The infusion was discontinued 6 h before surgery. The neurosurgical procedure was completed successfully without perioperative complications. Following confirmation of adequate hemostasis by the neurosurgical team, dual antiplatelet therapy with aspirin and ticagrelor was resumed on the first postoperative day. The patient remained clinically stable postoperatively (Figure 3).

**FIGURE 3:**
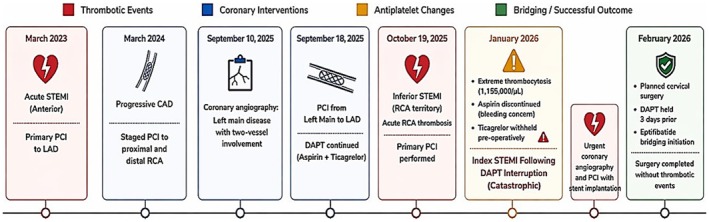
Clinical timeline of recurrent coronary events, antiplatelet interruption, and successful perioperative bridging strategy.

## 3 Methods (Differential Diagnosis, Investigations, and Treatment)

The clinical presentation of recurrent acute coronary syndromes in a patient with JAK2‐positive polycythemia vera raised a broad differential diagnosis, including stent thrombosis, progression of native coronary artery disease, hypercoagulability secondary to myeloproliferative neoplasm, and antiplatelet therapy interruption‐related thrombosis. Given the history of multiple prior percutaneous coronary interventions and extreme thrombocytosis, both mechanical (in‐stent thrombosis) and hematologic (PV‐related thrombotic propensity) etiologies were considered.

Diagnostic evaluation included serial electrocardiograms demonstrating evolving ST‐segment elevations, elevated cardiac biomarkers consistent with acute myocardial injury, and urgent coronary angiography, which confirmed culprit lesions corresponding to acute thrombotic occlusions in different coronary territories across events. Laboratory investigations consistently revealed persistent leukocytosis and marked thrombocytosis, with platelet counts exceeding 1,000,000/μL during the most recent admission.

Therapeutic management was guided by emergent revascularization strategies and hematologic risk modification. Primary percutaneous coronary intervention was performed during each acute presentation with successful restoration of coronary flow. Dual antiplatelet therapy was initially maintained with aspirin and ticagrelor; however, temporary discontinuation of antiplatelet agents prior to surgical procedures significantly contributed to thrombotic recurrence. Bridging antiplatelet therapy with intravenous eptifibatide was later implemented in the perioperative setting to balance bleeding and thrombotic risks.

We used the CARE reporting guideline [12] to draft this manuscript, and the CARE reporting checklist [13] during manuscript preparation and revision. The completed checklist is included as Supporting Information.

## 4 Conclusions and Results (Outcome and Follow‐Up)

This case illustrates the delicate and paradoxical balance between thrombosis and bleeding in patients with polycythemia vera complicated by extreme thrombocytosis and clinically suspected acquired von Willebrand syndrome. Interruption of dual antiplatelet therapy, particularly P2Y12 inhibitors, in the early post‐PCI period can lead to catastrophic thrombotic events. Conversely, this case also demonstrates that a carefully implemented bridging strategy using short‐acting intravenous antiplatelet agents may allow safe perioperative management in selected high‐risk patients requiring urgent non‐cardiac surgery. These findings underscore the critical importance of multidisciplinary coordination, individualized risk stratification, and optimized cytoreductive therapy, while highlighting the urgent need for prospective studies to establish evidence‐based perioperative antiplatelet management strategies in this vulnerable population.

## 5 Discussion

Polycythemia vera is a Philadelphia chromosome–negative myeloproliferative neoplasm characterized by clonal hematopoiesis driven by JAK2 V617F mutations. The disease is associated with hyperviscosity, leukocytosis, and thrombocytosis, resulting in a three‐ to five‐fold increased risk of arterial and venous thrombosis. Myocardial infarction, ischemic stroke, and venous thromboembolism remain leading causes of morbidity and mortality in affected patients [14].

In contrast, extreme thrombocytosis may paradoxically increase bleeding risk through the development of acquired von Willebrand syndrome. When platelet counts exceed 1,000,000/μL, excessive shear stress leads to proteolytic degradation of high–molecular‐weight vWF multimers. This process reduces ristocetin cofactor activity, impairs platelet adhesion, and predisposes patients to mucocutaneous hemorrhage despite a prothrombotic environment [15]. In patients undergoing PCI for STEMI, current guidelines recommend DAPT with aspirin and a P2Y12 inhibitor for at least 12 months to prevent stent thrombosis [16].

However, bleeding complications necessitate individualized risk assessment. European LeukemiaNet (ELN) recommendations allow temporary aspirin discontinuation in patients with AvWS, provided that aggressive cytoreductive therapy is instituted to reduce platelet counts. The ECLAP trial supports aspirin use in PV patients without active bleeding but does not address management in cases of extreme thrombocytosis complicated by AvWS [17].

In the present case, aspirin discontinuation two weeks before the index event was clinically justifiable given the presence of extensive cutaneous ecchymosis, extreme thrombocytosis, and clinically suspected acquired von Willebrand syndrome (AvWS). In contrast, interruption of ticagrelor two days prior to surgery, despite cardiology recommendations, was likely the precipitating factor for acute stent thrombosis. Early cessation of P2Y12 inhibition following drug‐eluting stent implantation is associated with a marked increase in thrombotic risk due to platelet rebound and heightened platelet reactivity [18, 19].

This mechanism plausibly explains the acute right coronary artery occlusion observed in our patient. This case highlights several critical clinical considerations. Patients with marked thrombocytosis and bleeding should undergo systematic evaluation for suspected acquired von Willebrand syndrome, including vWF antigen levels, ristocetin cofactor activity, and multimer analysis. Temporary aspirin discontinuation may be considered in selected cases of suspected or confirmed acquired von Willebrand syndrome but must be accompanied by rapid and effective cytoreduction. Interruption of P2Y12 inhibitors in the early post‐PCI period should be avoided whenever possible. When non‐cardiac surgery is unavoidable, bridging strategies using short‐acting intravenous antiplatelet agents should be considered. Notably, during the subsequent neurosurgical intervention, a bridging strategy using a short‐acting intravenous glycoprotein IIb/IIIa inhibitor (eptifibatide) was successfully implemented. This approach enabled temporary discontinuation of oral dual antiplatelet therapy while mitigating the risk of acute stent thrombosis. In contrast to the earlier event, the use of intravenous antiplatelet bridging was associated with a favorable perioperative outcome. Although randomized evidence remains limited, studies such as the BRIDGE trial have demonstrated that using short‐acting intravenous antiplatelet agents can maintain platelet inhibition without significantly increasing major bleeding risk in the perioperative setting [20].

Current expert consensus suggests that bridging strategies may be considered in selected high‐risk patients requiring urgent surgery, although decisions should be individualized [21].

## Author Contributions

**Behrouz zarei:** conceptualization, methodology, supervision, data curation, investigation, formal analysis, writing – review and editing, resources. **Amirhossein Rezaei Latifi:** conceptualization, data curation, investigation, writing – original draft, visualization, formal analysis, methodology, resources. **Mohammad Salari:** investigation, data curation, methodology, visualization.

## Funding

The authors have nothing to report.

## Ethics Statement

The authors have nothing to report.

## Consent

Written informed consent was obtained from the patient for publication of this case report.

## Conflicts of Interest

The authors declare no conflicts of interest.

## Supporting information

CARE Checklist.

## Data Availability

All data relevant to this case report are included in the article. Additional data are not publicly available to protect patient confidentiality but may be available from the corresponding author upon reasonable request.
