# Supplementary material for: A Therapeutic Dilemma: Catastrophic ST‐Elevation Myocardial Infarction Following Antiplatelet Therapy Interruption in a Patient With JAK2‐Positive Polycythemia Vera
Source: Clin Case Rep. 2026 Jul 29;14(8):e73264. doi: 10.1002/ccr3.73264 (PMC13420305; doi:10.1002/ccr3.73264)
Supplement: Supplementary file 1 — CARE Checklist. [file CCR3-14-e73264-s001.docx]

The CARE reporting checklist

|  | Item Description | Location (or reason for not reporting) |
| --- | --- | --- |
| **Sections** |  |  |
| [1. Title](https:/resources.equator-network.org/reporting-guidelines/care/items/title.html?utm_source=care&utm_medium=checklist&utm_campaign=CARE_2013_v1_1) |  | Title page |
| [2. Keywords](https:/resources.equator-network.org/reporting-guidelines/care/items/keywords.html?utm_source=care&utm_medium=checklist&utm_campaign=CARE_2013_v1_1) | The key elements of this case in 2–5 words. | After Abstract |
| [3. Abstract](https:/resources.equator-network.org/reporting-guidelines/care/items/abstract.html?utm_source=care&utm_medium=checklist&utm_campaign=CARE_2013_v1_1) | 3a – Introduction: What does this case add?  3b – Case presentation:   - The main symptoms of the patient(s). - The main clinical findings. - The main diagnoses and interventions. - The main outcomes.   3c – Conclusion: What are the main “take-away” lessons from this case? | Abstract  Abstract  Abstract, key clinical message |
| [4. Introduction](https:/resources.equator-network.org/reporting-guidelines/care/items/introduction.html?utm_source=care&utm_medium=checklist&utm_campaign=CARE_2013_v1_1) | Brief background summary of the case referencing the relevant medical literature. | Section 1 (Introduction), all paragraphs |
| [5a. Patient information](https:/resources.equator-network.org/reporting-guidelines/care/items/patient-information.html?utm_source=care&utm_medium=checklist&utm_campaign=CARE_2013_v1_1) | 5a – Demographic information of the patient (age, gender, ethnicity, occupation).  5b – Main symptoms of the patient (chief complaint).  5c – Medical, family, and psychosocial history—including lifestyle and genetic information whenever possible, details about relevant comorbidities, and past interv… | Section 2, paragraph 1  Section 2, paragraph 1  Section 2, paragraphs 1–2 |
| [6. Clinical findings](https:/resources.equator-network.org/reporting-guidelines/care/items/clinical-findings.html?utm_source=care&utm_medium=checklist&utm_campaign=CARE_2013_v1_1) | Describe the relevant physical examination (PE) findings. | Section 2, all clinical episodes |
| [7. Timeline](https:/resources.equator-network.org/reporting-guidelines/care/items/timeline.html?utm_source=care&utm_medium=checklist&utm_campaign=CARE_2013_v1_1) | Depict important date and times in this case (table or figure). | Section 2 + Figures 3 |
| [8. Diagnostic assessment](https:/resources.equator-network.org/reporting-guidelines/care/items/diagnostic-assessment-and-diagnosis.html?utm_source=care&utm_medium=checklist&utm_campaign=CARE_2013_v1_1) | 8a – Diagnostic methods (e.g., physical examination, laboratory testing, imaging, questionnaires)  8b – Diagnostic challenges (e.g., financial, language, or cultural)  8c – Diagnostic reasoning including other diagnoses considered  8d – Prognostic characteristics (e.g., staging) where applicable. | Section 3, paragraph 2 +figure 1-2  Section 3, paragraph 1  Section 3, paragraph 1  Section 5 (Discussion), paragraph 1 |
| [9. Therapeutic Intervention](https:/resources.equator-network.org/reporting-guidelines/care/items/therapeutic-interventions.html?utm_source=care&utm_medium=checklist&utm_campaign=CARE_2013_v1_1) | 9a – Types of intervention (e.g., pharmacologic, surgical, preventive, self-care)  9b – Administration (e.g., dosage, strength, duration)  9c – Changes in intervention (with rationale). | Section 2 + Section 3  Section 2, paragraphs 2–4  Section 2, last 2 paragraphs |
| [10. Follow up and outcomes](https:/resources.equator-network.org/reporting-guidelines/care/items/follow-up-and-outcomes.html?utm_source=care&utm_medium=checklist&utm_campaign=CARE_2013_v1_1) | 10a – Clinician and patient-assessed outcomes  10b – Important follow-up test results (positive and negative)  10c – Intervention adherence and tolerability (and how this was assessed)  10d – Adverse and unanticipated events. | Section 4 (Results/Conclusions)  Section 2 + Figures 1–3  Section 2, paragraph 1 & 4  Section 2, paragraphs 3–4 |
| [11. Discussion](https:/resources.equator-network.org/reporting-guidelines/care/items/discussion.html?utm_source=care&utm_medium=checklist&utm_campaign=CARE_2013_v1_1) | Discussion (including conclusion):  11a – Strengths and limitations of the management of this case  11b – Relevant medical literature  11c – Rationale for conclusions (including assessment of cause and effect)  11d – Main “take-away” lessons of this case report. | Section 5, Discussion  Section 5, references cited  Section 5, paragraph 4  Abstract conclusion + Discussion last paragraph |
| [12. Patient perspective](https:/resources.equator-network.org/reporting-guidelines/care/items/patient-perspective.html?utm_source=care&utm_medium=checklist&utm_campaign=CARE_2013_v1_1) | When appropriate patients should share their perspectives on the treatments they received. | Not obtained (emergency + ICU setting) |
| [13. Informed consent](https:/resources.equator-network.org/reporting-guidelines/care/items/informed-consent.html?utm_source=care&utm_medium=checklist&utm_campaign=CARE_2013_v1_1) | Did the patient give informed consent? Please provide if requested. | Patient consent statement section |
